# Supplementary material for: Thyme (Thymus quinquecostatus Celak) Polyphenol-Rich Extract (TPE) Alleviates HFD-Induced Liver Injury in Mice by Inactivating the TLR4/NF-κB Signaling Pathway through the Gut–Liver Axis
Source: Foods. 2023 Aug 16;12(16):3074. doi: 10.3390/foods12163074 (PMC10453248; doi:10.3390/foods12163074)
Supplement: Supplementary file 1 [file foods-12-03074-s001.zip › foods-2536120-supplementary.pdf]

Thyme (*Thymus quinquecostatus* Celak) Polyphenol-Rich Extract (TPE) Alleviates HFD-Induced Liver Injury in Mice by Inactivating the TLR4/NF-κB Signaling Pathway through the Gut–Liver Axis

Supplementary Materials

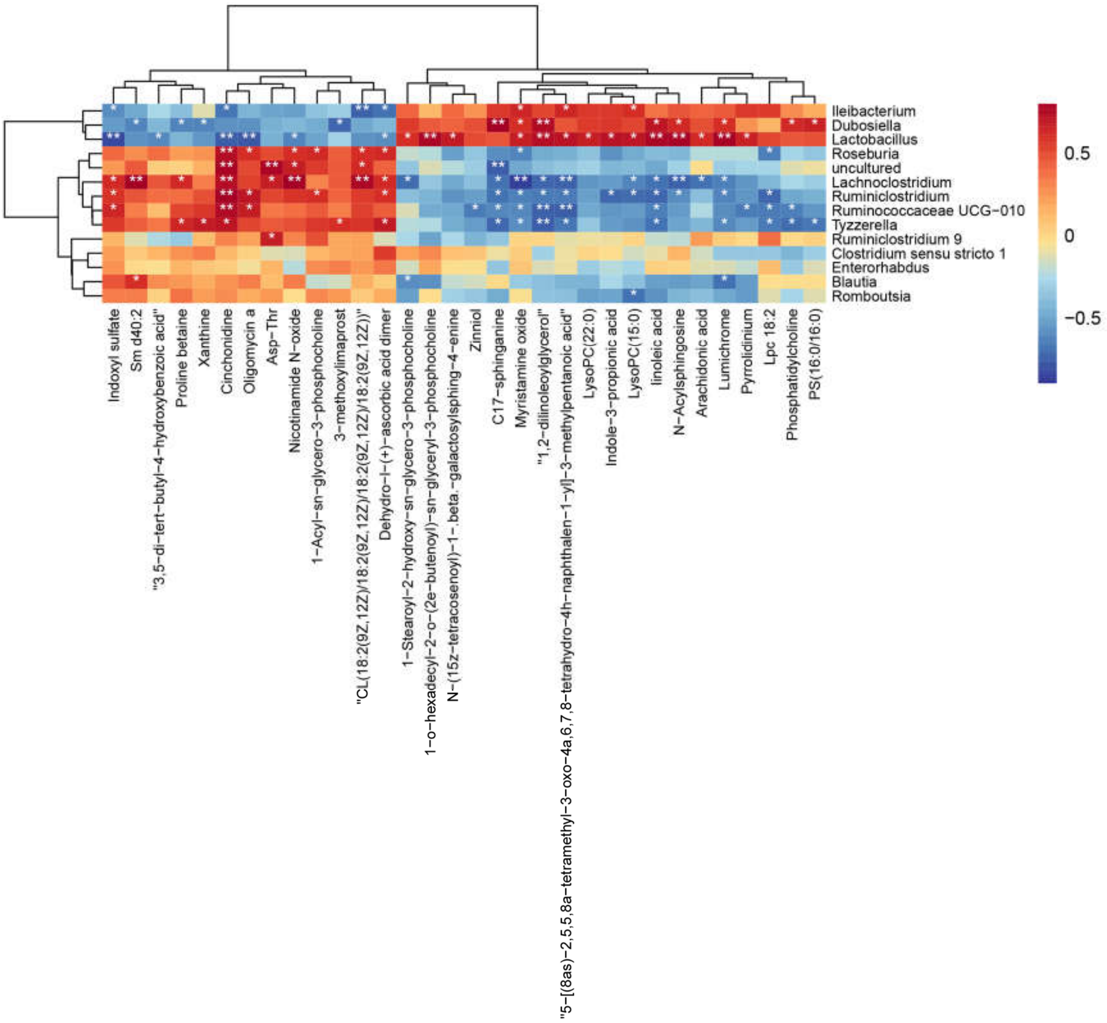

Figure S1. Correlations between serum metabolites of significant differences and the key gut microbial phylotypes were analyzed using Spearman's analysis (heatmap). The x-axis represents the differential metabolites in the serum. The y-axis represents the gut microbiota with differential abundance. **$p < 0.01$ between fecal metabolites and gut microbiota. *$p < 0.05$ between fecal metabolites and gut microbiota.

Table S1. Sequences of primers for RT-qPCR.

Target	Forward primer, reverse primer (5′→3′)
GAPDH	ACATCATCCCTGCATCCACT;<br>GTCCTCAGTGTAGCCCAAG
Pparα	CCTGGAAAGTCCCTTATCT;<br>GCCCTTACAGCCTTCACAT
Fasn	CCAAGCAGGCACACACAA;

	CACTCACACCCACCCAGA
Srebp-1	TGACCCGGCTATTCCGTGA; CTGGGCTGAGCAATACAGTTC
Hmgcr	AGCTTGCCCGAATTGTATGTG; TCTGTTGTGAACCATGTGACTTC
Cyp7a1	AGACGCACCTCGCTATTCTC; CACTCTGTAAAGCTCCACTC

Table S2. Identified compounds in the TPE by UPLC-MS/MS.

No.	tR (min)	Ion mode	CAS	Formula	Proposed compound
1	16.22	Negative	76822-21-4	$C_9H_{10}O_5$	Salvianic acid A
2	16.39	Negative	451-13-8	$C_8H_8O_4$	Homogentisic acid
3	18.21	Negative	1194-98-5	$C_7H_6O_3$	2,5-Dihydroxybenzaldehyde
4	18.31	Positive	531-75-9	$C_{15}H_{16}O_9$	Esculin
5	18.69	Positive	480-18-2	$C_{15}H_{12}O_7$	Taxifolin
6	18.96	Negative	327-97-9	$C_{16}H_{18}O_9$	Chlorogenic acid
7	19.15	Positive	5373-11-5	$C_{21}H_{20}O_{11}$	Cynaroside
8	20.06	Negative	530-57-4	$C_9H_{10}O_5$	Syringic acid
9	20.22	Positive	537-15-5	$C_{18}H_{16}O_8$	Rosmarinic acid
10	20.41	Positive	552-58-9	$C_{15}H_{12}O_6$	Eriodictyol
11	20.53	Negative	28608-75-5	$C_{21}H_{20}O_{11}$	Orientin
12	20.667	Negative	482-35-9	$C_{21}H_{20}O_{12}$	Quercetin-3-O-glucoside
13	20.96	Positive	7084-24-4	$C_{21}H_{20}O_{11}$	Kuromanin
14	21.21	Positive	13463-28-0	$C_{27}H_{32}O_{15}$	Eriocitrin
15	21.48	Positive	3681-93-4	$C_{21}H_{20}O_{10}$	Vitexin
16	21.71	Positive	27740-01-8	$C_{21}H_{18}O_{12}$	Scutellarin
17	22.23	Positive	17306-46-6	$C_{27}H_{30}O_{14}$	Rhoifolin
18	22.53	Negative	906-33-2	$C_{16}H_{18}O_9$	Neochlorogenic acid
19	22.54	Positive	480-10-4	$C_{21}H_{20}O_{11}$	Astragalin
20	22.6	Positive	38665-01-9	$C_{28}H_{32}O_{15}$	Neodiosmin
21	22.63	Positive	574-84-5	$C_{10}H_8O_5$	Fraxetin
22	22.72	Positive	28757-27-9	$C_{21}H_{20}O_{10}$	Apigenin-5-O-glucoside
23	22.88	Positive	520-36-5	$C_{15}H_{10}O_5$	Apigenin
24	22.89	Positive	29741-09-1	$C_{21}H_{18}O_{11}$	Apigenin 7-O-glucuronide
25	22.9	Positive	57378-72-0	$C_{25}H_{24}O_{12}$	Isochlorogenic acid C
26	23	Positive	115939-25-8	$C_{36}H_{30}O_{16}$	Salvianolic acid B
27	23.19	Positive	331-39-5	$C_9H_8O_4$	Caffeic acid
28	23.26	Positive	121521-90-2	$C_{36}H_{30}O_{16}$	Lithospermic acid B
29	23.27	Positive	480-40-0	$C_{15}H_{10}O_4$	Chrysin
30	24.24	Positive	529-53-3	$C_{15}H_{10}O_6$	scutellarin
31	24.48	Negative	537-98-4	$C_{10}H_{10}O_4$	Ferulic acid
32	24.63	Positive	480-36-4	$C_{28}H_{32}O_{14}$	Linarin
33	24.67	Negative	96574-01-5	$C_{26}H_{22}O_{10}$	Salvianolic acid A
34	25.02	Positive	-	$C_{26}H_{20}O_8$	Acacetin-7-O-glucuronide
35	25.53	Positive	491-70-3	$C_{15}H_{10}O_6$	Luteolin
36	25.62	Positive	117-39-5	$C_{15}H_{10}O_7$	Quercetin
37	25.82	Positive	51059-44-0	$C_{22}H_{20}O_{11}$	Wogonoside
38	27.37	Positive	480-41-1	$C_{15}H_{12}O_5$	Naringenin
39	27.86	Positive	520-18-3	$C_{15}H_{10}O_6$	Kaempferol
40	32.16	Positive	16545-23-6	$C_{18}H_{16}O_7$	Xanthomicrol
41	32.48	Positive	2957-21-3	$C_{16}H_{14}O_5$	Sakuranetin
42	32.83	Positive	437-64-9	$C_{16}H_{12}O_5$	Genkwanin
43	32.89	Positive	152743-19-6	$C_{18}H_{16}O_7$	Lysionotin
44	36.17	Positive	2174-59-6	$C_{20}H_{20}O_8$	5-O-Demethylnobiletin
45	38.18	Positive	2798-20-1	$C_{19}H_{18}O_7$	Gardenin B
46	47.14	Positive	77-52-1	$C_{30}H_{48}O_3$	Ursolic acid

Table S3. Quantitative Results of TPE.

Compound	Molecular Formula	Standard curve	R^2	Content (mg/g)
Scutellarin	$C_{21}H_{18}O_{12}$	y = 35872x − 242785	0.9968	231.56±1.41
Apigenin-7-O-glucuronide	$C_{21}H_{18}O_{11}$	y = 8184.4x + 120619	0.9939	52.1±0.79
Rosmarinic acid	$C_{18}H_{16}O_8$	y = 43162x − 405478	0.9974	206.28±1.12
Scutellarein	$C_{15}H_{10}O_6$	y = 4870.5x + 194906	−0.9965	68.18±0.55

Data are expressed as means ± SD (n = 3).

Qualitative and quantitative analysis of the main components of TPE

UPLC-MS/MS was used to identify the constituents of brown TPE powder. UPLC separation was conducted at 40°C with an Agilent SB-C18 column (2.1mm × 100mm, 1.8μm). The flow rate was 0.35 mL min^{-1}, the injection volume was 5 μL, and a binary gradient A and B were set as 0.1% formic acid aqueous solution and acetonitrile, respectively. The gradient program was set as follows: 0-10 min, 5% B; 10-20 min, 30% B; 20-25 min, 40% B; 25-30 min, 50% B; 30-40 min, 70% B; 40-45 min, 98% B; 45-60 min, 98% B; 60-65 min, 5% B; and 65-70 min, 5% B.

HPLC was used to quantify the major components of TPE. An Agilent C18 (2.1×100mm, 2.1μm) and a binary gradient comprised of 0.2% formic acid aqueous solution (A) and acetonitrile (B) were used to separate the components with a wavelength of 280 nm. The gradient elution as follows: 0-5 min, 5-10% B; 5-10 min, 10-10% B; 10-15 min, 10-18% B, 15-25 min, 18-26% B, 25-35 min, 26-35% B, 35-40 min, 35-18% B, 40-45 min, 18-10% B, 45-50 min, 10-5% B. Comparison of peak retention times between TPE sample and standard compounds to confirm peak identities. To quantify the main components of TPE, calibration curves of apigenin 7-O-glucuronide, scutellarin, rosmarinic acid and scutellarein were prepared, and compared with the area under the chromatographic peaks of TPE sample. The flow rate, sample injection and column temperature were set at 0.6 mL/min, 10μL, and 25°C, respectively.

Measurement of short-chain fatty acids (SCFAs)

Before SCFA analysis, frozen colonic contents (100mg) were homogenized in 0.5% phosphoric acid solution (1 mL) and centrifuged (17949g, 10 min, 4°C) to collect the supernatant. Afterward, the supernatant was treated with ethyl acetate (1:1, v/v) for 4 hours, then centrifuged (17949g, 10 min, 4°C) for a second time to collect the supernatant. The obtained supernatant was filtered through a PVDF membrane filter (0.22μm) and analyzed by GC-MS (Shimadzu, Japan). SCFA chromatographic separation was carried out using a DB-1701 capillary column (30 m x 0.25 mm x 0.25 m). The temperature program for the oven was as follows: initial temperature 35°C, holding 5 min; 15°C /min to 120°C; 5°C /min to 170°C; 20 °C /min to 250°C, holding 2 min.
